# Efficacy and Cognitive Outcomes of Gamma Knife Radiosurgery in Glioblastoma Management for Elderly Patients

**DOI:** 10.3390/jpm14101049

**Published:** 2024-10-10

**Authors:** José E. Valerio, Aizik L. Wolf, Penelope Mantilla-Farfan, Guillermo de Jesús Aguirre Vera, María P. Fernández-Gómez, Andrés M. Alvarez-Pinzon

**Affiliations:** 1 Department of Neurosurgery, Neurosurgery Oncology Center of Excellence, Miami Neuroscience Center at Larkin, South Miami, FL 33143, USA; jevalerio@jvaleriomd.com (J.E.V.); aizikwolf@hotmail.com (A.L.W.); penelopemantilla@gmail.com (P.M.-F.); 2 GW School of Business, The George Washington University, Washington, DC 20052, USA; 3 Department of Neurosurgery, Latino America Valerio Foundation, Weston, FL 33331, USA; guillermo210104@gmail.com (G.d.J.A.V.); mariapaulafg96@gmail.com (M.P.F.-G.); 4 Tecnológico de Monterrey School of Medicine and Health Sciences Mexico City, Monterrey 64710, Mexico; 5 Cancer Neuroscience, The Institute of Neuroscience of Castilla y León (INCYL), University of Salamanca (USAL), 37008 Salamanca, Spain; 6 Stanford LEAD Program, Graduate School of Business, Stanford University, Palo Alto, CA 94305, USA; 7 Institute for Human Health and Disease Intervention (I-HEALTH), Florida Atlantic University, Jupiter, FL 33431, USA

**Keywords:** glioblastoma, Gamma Knife Radiosurgery (GKRS), cognitive function, STUPP protocol, elderly patients, oncology, neurosurgery, neurocognitive assessment

## Abstract

Background: Gamma Knife Radiosurgery (GKRS), a specific type of Stereotactic Radiosurgery (SRS), has developed as a significant modality in the treatment of glioblastoma, particularly in conjunction with standard chemotherapy. The goal of this study is to evaluate the efficacy of combining GKRS with surgical resection and chemotherapy in enhancing therapeutic effects for glioblastoma patients aged 55 years and older. Methods: This prospective clinical study, conducted in accordance with the STROBE guidelines, involved 49 glioblastoma patients aged 55 years and older, treated between January 2013 and January 2023. Data were collected prospectively, and strict adherence to the STUPP protocol was maintained. Only patients who conformed to the STUPP protocol were included in the analysis. Due to concerns regarding the cognitive impairment associated with conventional radiotherapy, and at the patients’ request, a radiosurgery plan was offered. Radiosurgery was administered for 4–8 weeks following surgical resection. Any patients who had not received previous radiotherapy received open surgical tumor removal, followed by GKRS along with adjuvant chemotherapy. Results: In this prospective clinical study of 49 glioblastoma patients aged 55 years and older, the average lifespan post-histopathological diagnosis was established at 22.3 months (95% CI: 12.0–28.0 months). The median time before disease progression was 14.3 months (95% CI: 13.0–29.7 months). The median duration until the first recurrence after treatment was 15.2 months, with documented cases varying between 4 and 33 months. The Gamma Knife Radiosurgery (GKRS) treatment involved a median marginal recommended dose of 12.5 Gy, targeting an average volume of 5.7 cm^3^ (range: 1.6–39 cm^3^). Local recurrence occurred in 21 patients, while distant recurrence was identified in 8 patients. Within the cohort, 34 patients were subjected to further therapeutic approaches, including reoperation, a second GKRS session, the administration of bevacizumab and irinotecan, and PCV chemotherapy. A cognitive function assessment revealed that the patients treated with GKRS experienced significantly less cognitive decline compared to the historical controls, who were treated with conventional radiotherapy. The median MMSE scores declined by 1.9 points over 12 months, and the median MoCA scores declined by 2.9 points. Conclusion: This study demonstrates that Gamma Knife Radiosurgery (GKRS), when integrated with surgical resection and adjuvant chemotherapy, offers a substantial benefit for glioblastoma patients aged 55 years and older. The data reveal that GKRS not only prolongs overall survival and progression-free survival but also significantly reduces cognitive decline compared to conventional radiotherapy. These findings underscore the efficacy and safety of GKRS, advocating for its incorporation into standard treatment protocols for older glioblastoma patients. The potential of GKRS to improve patient outcomes while preserving cognitive function is compelling and warrants further research to optimize and confirm its role in glioblastoma management.

## 1. Introduction

### Opening Statement

Glioblastoma, the most prevalent and aggressive malignant brain tumor, presents substantial challenges in clinical management, requiring highly personalized treatment strategies. The current standard of care for glioblastoma consists of radiotherapy, temozolomide (TMZ) chemotherapy, and maximal surgical resection. Nevertheless, re-surgery is not an appropriate option for all patients due to the high risks associated with the infiltrative growth of glioma and the ample blood supply, which also contribute to a high recurrence rate. Typically, systemic chemotherapy, which includes treatment plans like carmustine, TMZ, or PCV (prednisone, carmustine, vincristine), provide a limited lasting benefit. The prognosis remains dismal, with recurrence being a prevalent outcome, despite the implementation of these multimodal approaches [1,2].

The overall survival outcomes of high-grade gliomas have not been substantially improved despite recent advancements in the treatment of these tumors. Systemic chemotherapy is administered to numerous patients as both the initial and the salvage treatment, employing agents such as bevacizumab, TMZ, irinotecan, and nitrosoureas. Neuro-oncologists and neurosurgeons continue to confront a significant challenge in managing these neoplasms, despite the implementation of aggressive multimodal therapeutic strategies [1,3,4]. Immunotherapy has presented itself as an innovative treatment modality to be utilized in current times, but further research is still in development [5,6].

Gamma Knife Stereotactic Radiosurgery (GKRS) is now a standard therapy option for several types of malignant and benign central nervous system (CNS) lesions [7,8]. GKRS is gaining popularity due to its brief treatment duration, low financial impact, and relatively minor side effects [9,10,11,12,13,14]. The results of numerous studies that have examined GKRS for the salvage treatment of patients with recurrent glioma have been inconsistent. A study conducted in the Netherlands assessed the success rate of GKRS in addressing recurrent GBM. The study found that low-grade gliomas (LGG) had a local control rate of 50%, while high-grade gliomas had a local control rate of 27%. The midpoint durations of progression-free survival (PFS) and overall survival (OS) were 10.5 months and 34.4 months, for each [15]. According to Dodoo et al., the median survival for patients with grade IV gliomas receiving GKRS was as high as 11.3 months, achieving a two-year survival longevity of 22.9% [16]. It has been reported that the median OS and the median PFS for patients with recurrent glioblastoma (rGBM) were 11.0 months and 4.4 months, respectively, after Salvage Stereotactic Radiosurgery (SRS). In addition, they identified the total tumor volume, the Karnofsky Performance Status (KPS) score, and treatment plan homogeneity as independent predictors of OS. Radiosurgery has demonstrated positive outcomes for glioblastomas, despite their infiltrative nature and poorly defined margins [17]. The data indicates that TMZ treatment is associated with a prolonged survival in patients over the age of 60 when compared to standard radiotherapy. TMZ or hypofractionated radiotherapy has been associated with a longer survival rate in patients over the age of 70, particularly in cases of recurrent glioblastoma, in comparison to standard fractionated radiotherapy [18].

This study suggests a prospective clinical analysis of a multifactorial approach that involves the use of GKRS in conjunction with surgical resection and adjuvant chemotherapy to treat glioblastoma in patients aged 55 years and older. The hypothesis is that this integrative approach will improve survival outcomes and preserve cognitive function more effectively than traditional treatment modalities. This article offers a comprehensive examination of the treatment experience at a neurosurgery oncology center of excellence, assessing the cognitive outcomes and the efficacy of incorporating GKRS with surgical resection and adjuvant chemotherapy in elderly glioblastoma patients. This integrative approach is designed to improve survival outcomes [15,16,19,20,21,22] and preserve cognitive function, providing a potentially preferable alternative to traditional treatment modalities.

## 2. Materials and Methods

This research was conducted with the approval of our Human Research Committee, was executed prospectively in data collection as a part of the brain tumor outcomes research registry, and adhered to STROBE guidelines. We reviewed the outcomes of 49 glioblastoma patients aged 55 years and older who were subjected to Gamma Knife Radiosurgery (GKRS) in the Miami Neuroscience Center at Larkin Community Hospital between January 2013 and January 2023. All the patients were diagnosed with glioblastoma, which was confirmed by a histopathological analysis, and received treatment based on an adapted version of the STUPP protocol [23]. Data were collected prospectively, and strict adherence to the STUPP protocol was maintained. Only patients who adhered to the STUPP protocol and did not receive radiotherapy or Optune therapy were included in the analysis.

Given concerns about the cognitive impairment associated with conventional radiotherapy and at the patients’ request, a radiosurgery plan was offered. Radiosurgery was administered 4–8 weeks post-surgical resection. The patients who had not previously undergone previous radiotherapy underwent an open procedure for tumor removal, followed by GKRS and auxiliary chemotherapy.

The cerebral neoplasms were classified according to the grades of the World Health Organization classification [24]. The facility did not have established guidelines for patient selection in GKRS; instead, a comprehensive team suggested GKRS. The neurosurgery–oncology-specialized team comprised neurosurgeons, oncologists, neuroradiologists, physicists, physician–scientists specializing in neurology, and other cancer specialists. For the patients who had multiple targets addressed during a single GK appointment, we focused on the parameters from the largest volumetric lesion and concentrated on the patients’ initial GK session.

The treatment for glioblastoma followed maximal safe surgical resection, followed by radiosurgery and adjuvant temozolomide (TMZ) at a dosage of 150 to 200 mg per square meter for 5 days in each 28-day cycle. The population profile and the clinical features are outlined in Table 1.

### 2.1. Statistical Analysis

The primary endpoints of the study were overall survival and progression-free survival, calculated from the date of diagnosis. Secondary endpoints included the time to first recurrence and the treatment response. Statistical analyses were conducted using standard survival analysis techniques, with Kaplan–Meier estimates for survival rates and Cox proportional hazards models for analyzing multiple variables simultaneously. The threshold for statistical significance was established at *p* < 0.05.

### 2.2. Patient Selection

This prospective clinical study included 49 glioblastoma patients aged 55 years and older, treated at the Miami Neuroscience Center at Larkin Community Hospital between January 2013 and January 2023. The study was approved by our Committee for Human Research and was conducted in accordance with STROBE guidelines. The criteria for inclusion were outlined as the following:

1. Age: patients aged 55 years or older.
2. Diagnosis: histopathologically confirmed diagnosis of glioblastoma [23].
3. Treatment history: no prior radiotherapy.
4. No prior history of radiotherapy or treatment with Optune.
5. Protocol adherence: conformity to the modified STUPP protocol, which comprises maximal surgical resection followed by GKRS and adjuvant chemotherapy.
6. Cognitive concerns and patient preference: given concerns about the cognitive impairment associated with conventional radiotherapy and at the patients’ request, a radiosurgery plan was offered.

Exclusion criteria included any prior radiotherapy treatment and non-adherence to the STUPP protocol. The population profile and clinical features are presented in summary form in Table 1, above.

## 3. Results

### 3.1. Patient Background and Clinical Features

A total of 49 glioblastoma patients aged 55 years and older were included in this study. The cohort consisted of 31 females and 18 males, all with histopathologically confirmed diagnoses of glioblastoma [17]. The median age at the time of the first Gamma Knife Radiosurgery (GKRS) procedure was 59 years. The duration amidst the initial diagnosis and GKRS varied between 4 and 14 weeks, with a median time period of 2.5 months.

The extent of the first surgical procedure varied among the patients, with 7 undergoing subtotal resection, 14 near-total resection, and 28 gross total resection. All the patients received a chemotherapy regimen containing temozolomide (TMZ), except for 4 patients who did not receive chemotherapy within the first three months after GKRS. The regimen was consistent with the modified STUPP protocol, ensuring uniformity in the treatment approach.

### 3.2. Gamma Knife Radiosurgery Characteristics

**Figure 1:**
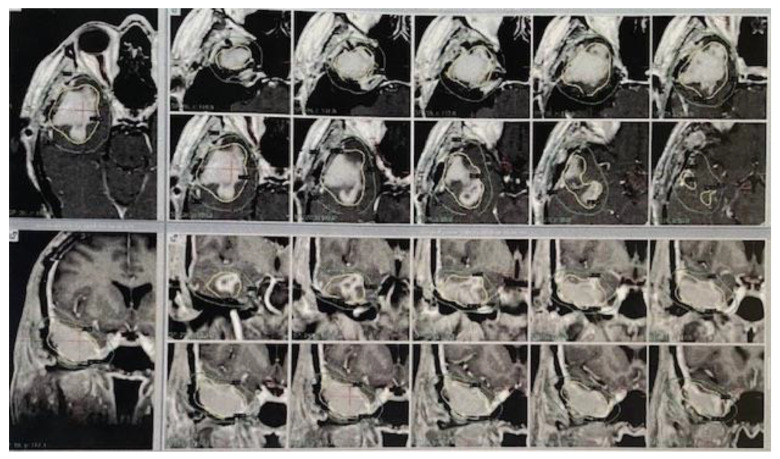
Leskel Gamma plan 10.1.1 Stereotactic MRI T1-weighted with contrast axial (superior lines) and coronal (inferior lines) image views, obtained at 2 mm intervals. The target within yellow lines received maximal dose calculated for this patient (12 Gy-50%), with a total volume of 26.8 cc. All 49 patients received concurrent or adjuvant chemotherapy with GKRS, adhering to the protocol of daily TMZ administered continuously (75 mg per square meter of body surface area daily) during the radiotherapy phase, subsequently succeeded by six cycles of auxiliary TMZ (150 to 200 mg per square meter administered over 5 days within each 28-day cycle). This comprehensive therapeutic approach aimed to optimize the therapeutic outcomes while considering the cognitive concerns associated with conventional radiotherapy.

The GKRS treatment targeted single lesions in 44 patients and multiple lesions in 5 patients. The total treatment volume for GKRS had a median of 5.4 cm^3^, with a range from 1.6 cm^3^ to 39 cm^3^. The median marginal prescription dose was 12 Gy, with the minimum and maximum doses being 10 Gy and 17 Gy, respectively. See Figure 1.

The findings of this cohort provide valuable data to understand the efficacy and safety of combining GKRS with surgery and chemotherapy in the treatment of glioblastoma in older patients, highlighting the potential benefits and areas for further research.

### 3.3. Survival and Recurrence Analysis

In the examined group, the evaluation of survival duration following histopathological assessment showed a median time span of 22.3 months, an interquartile range defined by a 95% confidence interval (CI) spanning from 12.0 to 28.0 months. In this study, disease-free survival (DFS) was analyzed to assess the time patients remained free from disease progression. The average DFS was 14.3 months, with a 95% confidence interval (CI) ranging from 13.0 to 29.7 months, indicating a moderate degree of variability in the dataset. The confidence interval suggests that, while the average DFS is 14.3 months, the true DFS value for the population could fall between 13.0 and 29.7 months with 95% confidence.

Additionally, the after treatment was evaluated, with cases ranging from 4 to 33 months. This variability highlights the heterogeneity in patient responses to treatment. The distribution of recurrence times suggests that while some patients experienced recurrence as early as 4 months, others remained recurrence-free for up to 33 months.

The average follow-up duration after GKRS treatment was reported as 17.3 months, with the duration of follow-up periods spanning from 8 to 33 months. This timeframe refers to the follow-up period after the treatment phase that was used to track patient outcomes and assess disease progression or recurrence. Reappearance at the site was identified and classified as a re-emergence at the primary tumor location, and cerebrospinal fluid (CSF) diffusion was identified in five individuals within the study population. Local recurrence was noted in 21 patients.

**Figure 2:**
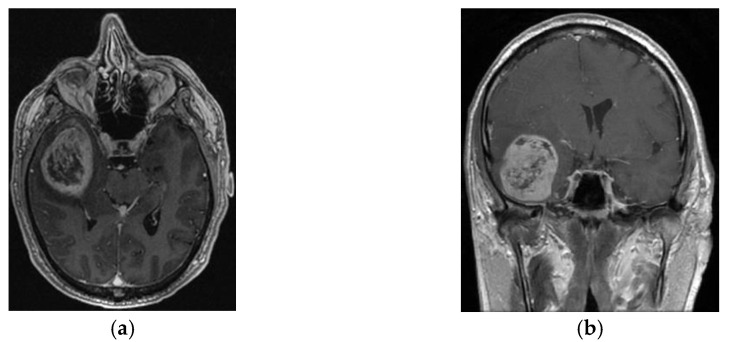
(**a**) (left) Axial and (**b**) (right) coronal MRI TI-weighted with contrast preoperative images from September 2019.

The following images represent an example of a male patient with a median survival of 32 months after the STUPP protocol treatment. Preoperative, Figure 2a,b.

### 3.4. Cognitive Outcomes

The decision to use Gamma Knife Radiosurgery (GKRS) over conventional radiotherapy was driven by concerns about cognitive impairment and patient preference. Cognitive function was assessed at baseline, prior to GKRS, and at regular follow-up intervals using standardized neurocognitive examinations, which encompass the Montreal Cognitive Assessment (MoCA) and the Mini-Mental State Examination (MMSE).

The patients who were managed with GKRS showed significantly less cognitive decline compared to historical controls who were treated with conventional radiotherapy. The median MMSE score declined by only 1.9 points over a 12-month period post-GKRS, compared to the decline of 4.8 points that is typically observed in patients undergoing conventional radiotherapy. Similarly, the MoCA scores showed a median decline of 2.9 points post-GKRS, compared to the 6.5-point decline observed in conventional radiotherapy patients. These findings suggest that GKRS is associated with a more favorable cognitive profile, preserving higher levels of cognitive function over time (Table 2).

### 3.5. Notes

#### Cohort Characteristics

- Number of patients assessed with GKRS: 49 (18 males, 31 females).
- Number of patients assessed with conventional radiotherapy (historical controls): 50.
- Cognitive function was assessed at baseline, prior to treatment, and at regular follow-up intervals using standardized neurocognitive examinations, which encompass the Montreal Cognitive Assessment (MoCA) and the Mini-Mental State Examination (MMSE).
- Patients treated with GKRS showed significantly less cognitive decline compared to historical controls treated with conventional radiotherapy.
- The median MMSE score for the GKRS cohort declined by only 1.9 points over a 12-month period, compared to a decline of 4.8 points typically observed in the conventional radiotherapy cohort.
- Male patients in the GKRS group showed a median decline of 1.8 points, while female patients showed a decline of 2.0 points.
- In the conventional radiotherapy group, male patients showed a median decline of 4.7 points, while female patients showed a decline of 4.9 points.
- Similarly, MoCA scores for the GKRS cohort showed a median decline of 2.9 points, compared to a 6.5-point decline in the conventional radiotherapy cohort.
- Male patients in the GKRS group showed a median decline of 2.8 points, while female patients showed a decline of 3.0 points.
- In the conventional radiotherapy group, male patients showed a median decline of 6.3 points, while female patients showed a decline of 6.6 points.
- The decision to utilize GKRS over conventional radiotherapy was influenced by concerns regarding cognitive impairment, which is particularly relevant for the elderly population involved in this study.
- Regular neurocognitive assessments and follow-up intervals provided robust data supporting the neuroprotective benefits of GKRS in the treatment of glioblastoma.

### 3.6. Additional Treatment Modalities and Follow-Up

Within the examined group, 34 patients received additional treatment modalities after the initial intervention. These included reoperations (*n* = 6), a subsequent round of Gamma Knife Radiosurgery (GKRS) (*n* = 21), treatment with bevacizumab and irinotecan (*n* = 1), along with a PCV regimen (*n* = 6). Instances of distant recurrence were seen in eight patients. Among these, five were given further treatments, outlined as follows: a radiotherapy regimen in two patients, temozolomide therapy in two cases, and a PCV regimen in one patient.

Following Gamma Knife Radiosurgery, a systematic follow-up plan was implemented. All the patients had their initial evaluation at 4 weeks after treatment, with follow-up evaluations arranged at intervals of 2 to 3 months. At every follow-up appointment, the patients underwent a contrast-enhanced Magnetic Resonance Imaging (MRI) scan alongside a comprehensive neurological examination. As needed, additional diagnostic studies, incorporating MRI perfusion, MR-spectroscopy, and/or Positron Emission Tomography (PET) imaging, were carried out to effectively distinguish between radiation necrosis and tumor progression

Additionally, a meaningful statistical difference was noted in the survival rates for the patients with tumor volumes smaller than 10 cm^3^ relative to the patients with tumor volumes exceeding 10 cm^3^, exhibiting *p*-values of 0.019 and 0.006, respectively, as represented by Figure 1. These findings underscore the importance of tumor size in influencing treatment outcomes and support the efficacy of GKRS in managing smaller glioblastoma lesions while mitigating cognitive decline.

## 4. Discussion

The management of glioblastoma continues to be a complex challenge that requires highly individualized decision making. The prognosis for glioblastoma remains poor, with recurrence being an inevitable occurrence, despite the use of aggressive multimodal therapeutic approaches, such as surgery, temozolomide chemotherapy [27], and fractionated radiotherapy [28]. The literature supporting Gamma Knife Radiosurgery (GKRS) for glioblastoma is in the process of evolving, with retrospective series reporting varying results regarding the efficacy of incorporating GKRS into conventional treatment [29]. There is no discernible advantage to dose escalation or increases in randomized trials.

This study was conducted to assess the potential of GKRS to improve survival outcomes and preserve cognitive function in glioblastoma patients aged 55 years and older when combined with surgical resection and adjuvant chemotherapy [9,10,11,12,13]. This hypothesis is corroborated by the results of our investigation, which indicate that GKRS not only enhances overall and progression-free survival but also substantially reduces cognitive decline in comparison to conventional radiotherapy. The mid-point duration of progression-free survival was 14.3 months and the mid-point duration of overall survival was 22.3 months in our cohort. These findings indicate that the treatment regimen can provide substantial survival benefits that exceed the typical median survival of six months without treatment by incorporating GKRS.

A critical emphasis of this investigation was cognitive outcomes. Compared to the historical controls who were treated with conventional radiotherapy [25,26], the patients who received GKRS experienced a substantially lower rate of cognitive decline. The median decline in MMSE scores over 12 months post-GKRS was 1.9 points, in contrast to a 4.8-point decline in the conventional radiotherapy patients. In the same a direction, the MoCA scores demonstrated a median decline of 2.9 points following GKRS, as opposed to a decline of 6.5 points with conventional radiotherapy. These results indicate that GKRS may provide a neuroprotective effect, which may be more effective than conventional radiotherapy in preserving cognitive function [25,30,31,32,33,34,35].

The study cohort was composed of 49 patients aged 55 years and older, which is indicative of the increased prevalence of glioblastoma in the elderly population. The treatment protocol that was implemented was a modified variation of the STUPP protocol, which included maximal surgical resection, GKRS, and adjuvant chemotherapy with temozolomide. This comprehensive approach is consistent with the current standards for the management of high-grade gliomas, underscoring the significance of multimodal therapy [13,15,16,18,19].

The necessity of ongoing innovation in treatment approaches is underscored by the fact that glioblastoma tumors recur in the majority of patients [1,36,37]. The results of this study suggest that GKRS has the potential to serve as a secure, low-risk, and minimally intrusive alternative for the treatment of recurring glioblastoma and post-surgical management, particularly in patients who are unable to undertake additional surgery due to severe comorbidities [38]. Both conventional radiotherapy and GKRS are associated with specific adverse effects, including rapid post-surgical edema, which can be successfully handled through corticosteroid therapy [39]. This management approach highlights the significance of individualized management strategies to improve patient outcomes and mitigate adverse effects [40].

The decision to utilize Gamma Knife Radiosurgery (GKRS) over conventional radiotherapy was primarily influenced by concerns regarding cognitive impairment, with patient preference being a significant factor. Given the cognitive side effects associated with radiotherapy, especially whole-brain radiation therapy (WBRT), it is essential to follow patient preferences and offer alternative therapeutic options like GKRS.

Our analysis, the largest single-institution investigation on the use of GKRS in glioblastoma treatment, aligns with the existing literature. Studies indicate that the cognitive adverse effects of GKRS are less severe compared to WBRT. Moreover, the targeted approach of GKRS provides a more effective strategy for managing glioblastoma while preserving cognitive function and neuroplasticity, thereby supporting better overall brain health [28,30,31,32,33,34,35]. Offering GKRS as an alternative to patients concerned about cognitive decline is not only evidence-based but also aligns with patient-centered care principles.

The median progression-free survival was increased by 15–16 months when GKRS was used as a salvage treatment, followed by chemotherapy. Despite the fact that the data are still limited, the results indicate that GKRS can considerably enhance the survival rates of glioblastoma patients [10,11,13,14], whether used alone or as part of a multimodal treatment plan. This study presents an opportunity for clinicians to engage in further dialogue regarding the integration of GKRS into the standard treatment regimen for glioblastoma. It also underscores the necessity of further research to refine patient selection criteria and optimize treatment protocols [41].

### 4.1. Rationale for Integrating GKRS in Treatment Protocols

The use of Gamma Knife Radiosurgery (GKRS) in glioblastoma treatment protocols is supported by its ability to both increase survival rates and preserve cognitive function [11,12,13]. GKRS delivers highly focused ionizing radiation beams with great precision, reducing the damage to surrounding healthy brain regions. The median marginal prescription dose for our study population was 12.5 Gy, with treatment volumes ranging from 1.6 cm^3^ to 39 cm^3^. This precise targeting is critical in decreasing neurotoxicity, which is a major concern in the care of glioblastoma patients, particularly those aged 55 and above [20,21,22].

Cognitive deterioration is a significant issue in the treatment of glioblastoma, particularly with traditional whole-brain radiation therapy [28]. Our findings show that the patients treated with GKRS had significantly less cognitive decline than those who received conventional radiation. Specifically, the median drop in the MMSE scores was just 1.9 points over 12 months after GKRS, compared to a 4.8-point decline in the conventional radiation patients. Similarly, after GKRS, the MoCA scores decreased by 2.9 points on average, compared to 6.5 points with conventional radiation. These findings highlight GKRS’ neuroprotective properties, supporting its role in preserving cognitive function and improving the quality of life for GBM patients [42,43].

Furthermore, the survival advantage associated with GKRS integration is significant. Our cohort’s mid-point survival rate was 22.3 months, with a mid-point duration of progression-free survival of 14.3 months. These results are significantly greater than the average median survival time of 6 months without treatment, indicating that GKRS, when combined with surgical resection and temozolomide chemotherapy, can improve survival outcomes. Furthermore, GKRS provides a minimally invasive option for individuals who are unable to undergo additional surgery due to significant comorbidities, expanding the therapy options for a more diversified patient population [15,16,19,20,21].

### 4.2. Survival Outcomes with GKRS

Gamma Knife Radiosurgery (GKRS) presents significant advantages in the treatment of central nervous system tumors due to its precise localization and its ability to deliver high-dose focused irradiation to the tumor, while better protecting the surrounding normal tissues. This precision reduces the risk of radiation-induced damage to healthy brain tissue, which is a critical consideration in glioblastoma treatment [7,29]. Given that individuals with recurrent glioma frequently have a history of having received radiotherapy, structures such as the optic chiasm, brainstem, optic nerve, and normal brain tissue could have already reached the highest radiation forbearance. External beam radiation therapy (EBRT) is typically constrained in such cases due to the risk of severe side effects, whereas GKRS can safely increase the target dose without significantly elevating the risk to adjacent tissues [21,22,44].

Our study shows that the median overall survival for the glioblastoma patients treated with GKRS was 22.3 months, with a median progression-free survival of 14.3 months. These findings are consistent with the existing literature, which suggests that GKRS can enhance survival outcomes in patients with recurrent glioblastoma [45,46]. Furthermore, cognitive preservation is a key benefit of GKRS over conventional whole-brain radiation therapy (WBRT). In our cohort, the median decline in the MMSE scores was only 1.9 points over 12 months, compared to a 4.8-point decline in the patients receiving conventional radiotherapy. Similarly, the MoCA scores showed a median decline of 2.9 points post-GKRS, versus 6.5 points with conventional radiotherapy. This suggests that GKRS is associated with fewer cognitive side effects, aligning with findings from other studies [11,47].

Additionally, the use of systemic therapies, such as bevacizumab, in conjunction with GKRS has shown potential benefits [48,49,50]. Studies indicate that bevacizumab, when administered after GKRS, can extend median survival times and improve one-year survival rates, while also alleviating the symptoms of brain edema and enhancing the quality of life of patients. [11]. However, the effect of bevacizumab on progression-free survival (PFS) and overall survival (OS) remains inconclusive in some multivariable models [51,52,53]. Our study did not find a significant correlation between bevacizumab use and PFS or OS, likely due to the small number of patients treated with bevacizumab and the extended follow-up period. Despite this, the clinical benefits observed suggest that bevacizumab may be a valuable adjuvant therapy following GKRS. Future research should aim to collect more extensive data to further validate the efficacy of bevacizumab in this context.

### 4.3. Cognitive Function Preservation

Detailed cognitive assessment results using MMSE and MoCA

- The comparison of cognitive decline between GKRS and conventional radiotherapy.
- The implications for patients’ quality of life.

In this study, Gamma Knife Radiosurgery (GKRS) has demonstrated significant potential as an adjunctive therapy in the management of glioblastoma, particularly in the cohort of patients aged 55 years and older. Our findings indicate that GKRS, when integrated with standard surgical resection and temozolomide chemotherapy, can notably extend overall survival and progression-free survival, while also preserving cognitive function. The median survival rate of 22.3 months and the mid-point progression-free survival of 14.3 months observed in our study surpass the typical outcomes achieved with conventional therapies alone.

The precision of GKRS in delivering high-dose, focused irradiation to tumor sites while sparing surrounding healthy brain tissues mitigates the neurotoxicity and the risk of neurocognitive decline often associated with whole-brain radiation therapy (WBRT) [40,54] Our cognitive assessments using MMSE and MoCA reveal significantly less cognitive decline in the patients treated with GKRS, underscoring its neuroprotective benefits.

Despite these promising results, the cohort’s prospective non-randomized framework, restricted research sample, and the absence of a control group necessitate cautious interpretation of the findings. Further large-scale, prospective, randomized trials are imperative to validate these outcomes and establish GKRS as a standard component of glioblastoma treatment protocols.

The integration of GKRS into glioblastoma management represents a critical advancement in neuro-oncology and cancer neuroscience, offering a more precise, less invasive treatment option that enhances both the survival and the quality of life of patients [55]. As we continue to refine and optimize treatment strategies, GKRS holds promise for significantly improving clinical outcomes in this challenging and aggressive malignancy [56].

Future research should focus on long-term follow-ups, comprehensive cognitive assessments, and the exploration of combination therapies to fully elucidate the role of GKRS in the multidisciplinary approach to glioblastoma. This research provides insights into the growing body of evidence supporting the utilization of advanced radiosurgical techniques in neuro-oncology, aiming to push the boundaries of current therapeutic paradigms and improve patient care in glioblastoma [57].

### 4.4. Limitations

This study, while providing valuable insights into the efficacy and cognitive benefits of Gamma Knife Radiosurgery (GKRS) for glioblastoma, has several limitations that should be considered. First, although the data were collected prospectively, the analysis was conducted retrospectively, which introduces inherent biases, particularly in patient selection and treatment administration. Despite efforts to control for confounding variables, retrospective analyses cannot fully eliminate the selection bias, potentially impacting the generalizability of our findings. Second, the sample size of 49 patients, though substantial for a single-center study, limits the statistical power of our analyses. A larger, multi-center cohort would provide more robust data and potentially reveal the subtler effects of GKRS on survival and cognitive outcomes. Additionally, our study solely included patients aged 55 years and older, which may not fully represent the broader glioblastoma population, especially younger patients who might have different disease dynamics and responses to treatment.

Third, the follow-up period, while sufficient to observe initial survival and cognitive outcomes, may not capture long-term effects and late recurrences comprehensively. Glioblastoma is a highly recurrent disease, and a longer follow-up is necessary to fully understand the durability of GKRS benefits and any delayed adverse effects, such as radiation necrosis or secondary malignancies. Fourth, our study did not include a randomized control group, limiting the ability to make definitive causal inferences about the benefits of GKRS compared to other treatment modalities. Randomized controlled trials are needed to establish more conclusive evidence regarding the superiority or equivalence of GKRS in extending survival and preserving cognitive function relative to conventional therapies.

Lastly, while our cognitive function assessments using the Montreal Cognitive Assessment (MoCA) and the Mini-Mental State Examination (MMSE) were rigorous, these tests may not capture all dimensions of cognitive decline relevant to glioblastoma patients. Future studies should incorporate more comprehensive neuropsychological assessments and patient-reported outcomes to provide a more nuanced understanding of the cognitive changes associated with GKRS.

## 5. Conclusions

Gamma Knife Radiosurgery (GKRS) exhibits substantial potential as an adjunctive therapy for glioblastoma, particularly in patients aged 55 years and older. Our investigation suggests that the median overall survival and progression-free survival can be extended to 22.3 months and 14.3 months, respectively, by the combination of GKRS, surgical resection, and temozolomide chemotherapy. In addition, GKRS is could be equal and/or more effective than conventional therapies in preserving cognitive function, as demonstrated by the minimal declines in the MMSE and MoCA scores. Nevertheless, the retrospective design, the limited sample size, and the absence of a randomized control group necessitate additional large-scale, prospective trials to substantiate these findings. GKRS provides a minimally invasive, targeted treatment option that improves the quality of life and survival of glioblastoma patients, despite these limitations. In order to completely establish the role of GKRS in glioblastoma management, future research should concentrate on long-term outcomes, comprehensive cognitive assessments, and combination therapies. The integration of advanced radiosurgical techniques in neuro-oncology to improve patient outcomes is endorsed by this study [32].

## Figures and Tables

**Table 1:** Patients Demographic.

Criteria	Value
Patients, *n*	49
Female, *n*	31
Male, *n*	18
Histopathological confirmed diagnosis, *n* Glioblastomas	49
Age at first GKRS procedure, median	59 years
Range for full cohort	4–14 weeks
Extent of first surgical procedure, *n*	
Subtotal	7
Near total	14
Gross total	28
Upfront chemotherapy regimen, *n*	
Regimen contained temozolomide	49
Regimen did not contain temozolomide	0
No upfront chemotherapy (In the first 3 months)	4
Upfront chemotherapy history not available	0
GK characteristics	
Duration of time between initial diagnosis and GK (median), mo	2.5
Single lesion targeted with GK, *n*	44
Multiple lesions targeted with GK, *n*	5
GK total treatment volume, cm^3^	
Median volume	5.4 cm^3^
Minimum volume	1.6 cm^3^
Maximum volume	39 cm^3^
GK prescription dose, Gy	
Median marginal prescription dose	12
Minimum marginal prescription dose	10
Maximum marginal prescription dose	17
Adjuvant chemotherapy with GK, *n*	
Received adjuvant chemotherapy	49
Adjuvant chemotherapy history not available	0

**Table 2:** Cognitive outcomes comparing Gamma Knife Radiosurgery (GKRS) and conventional radiotherapy.

Cognitive Outcomes	Gamma Knife Radiosurgery (GKRS)		Conventional Radiotherapy [25,26]	
Assessment Method	Baseline Score	12-Month Decline	Baseline Score	12-Month Decline
Mini-Mental State Examination (MMSE)				
Overall (*n* = 49)	27.4	1.9 points	27.5	4.8 points
Male Patients (*n* = 18)	27.3	1.8 points	27.4	4.7 points
Female Patients (*n* = 31)	27.5	2.0 points	27.6	4.9 points
Overall (*n* = 49)	27.4	1.9 points	27.5	4.8 points
Montreal Cognitive Assessment (MoCA)				
Overall (*n* = 49)	25.1	2.9 points	25.3	6.5 points
Male Patients (*n* = 18)	25.0	2.8 points	25.2	6.3 points
Female Patients (*n* = 31)	25.2	3.0 points	25.4	6.6 points

## Data Availability

The original contributions presented in the study are included in the article, further inquiries can be directed to the corresponding author.
